# Robot-Assisted Navigation versus Computer-Assisted Navigation in Primary Total Knee Arthroplasty: Efficiency and Accuracy

**DOI:** 10.1155/2013/794827

**Published:** 2013-06-24

**Authors:** Tanner C. Clark, Frank H. Schmidt

**Affiliations:** ^1^University of Washington School of Medicine, 1959 NE Pacific Street, Seattle, WA 98195, USA; ^2^Big Horn Basin Bone and Joint, 720 Lindsay Lane, Suite C, Cody, WY 82414, USA

## Abstract

*Background*. Since the introduction of robot-assisted navigation in primary total knee arthroplasty (TKA), there has been little research conducted examining the efficiency and accuracy of the system compared to computer-assisted navigation systems. *Objective*. To compare the efficiency and accuracy of Praxim robot-assisted navigation (RAN) and Stryker computer-assisted navigation (CAN) in primary TKA. *Methods*. This was a retrospective study consisting of 52 patients who underwent primary TKA utilizing RAN and 29 patients utilizing CAN. The primary outcome measure was navigation time. Secondary outcome measures included intraoperative final mechanical axis alignment, intraoperative robot-assisted bone cut accuracy, tourniquet time, and hospitalization length. *Results*. RAN navigation times were, on average, 9.0 minutes shorter compared to CAN after adjustment. The average absolute intraoperative malalignment was 0.5° less in the RAN procedures compared to the CAN procedures after adjustment. Patients in the RAN group tended to be discharged 0.6 days earlier compared to patients in the CAN group after adjustment. *Conclusions*. Among patients undergoing TKA, there was decreased navigation time, decreased final malalignment, and decreased hospitalization length associated with the use of RAN when compared to CAN independent of age, BMI, and pre-replacement alignment.

## 1. Introduction

Technological advancements have revolutionized the field of orthopedics. Robot-assisted navigation (RAN) in partial and total knee arthroplasty (TKA) now enables surgeons to execute these procedures with unprecedented accuracy and precision [1–4]. Osteoarthritis is the most common joint disorder in the United States and the aging US population is expected to grow substantially. According to an article by Iorio et al. [5], the authors reported that during the period of 2000 to 2030, the elderly population is expected to increase 104%, accompanied by a projected 565% increase of primary TKA procedures. Currently, the annual total expenditure for TKA is approximately $18.75 billion. In addition, higher BMIs are associated with increased surgical time of TKA [6], which may decrease operating room efficiencies. This tremendous increase of TKA will intensify the demand for competent physicians and accommodating a heavy workload while maintaining quality standards may be achieved through the use of navigation systems. 

Computing power has exponentially increased during the past decade and is utilized in the area of joint reconstruction. Computer-assisted navigation (CAN) has been shown to significantly improve implant alignment in TKA compared to conventional techniques [7–13]. A study by Choong et al. demonstrated that there is a correlation between optimal alignment and improved quality of life and knee functionality [12]. Studies have also demonstrated that proper alignment of the prosthesis during total knee replacement is critical in maximizing implant survival [11, 13]. 

The implementation of CAN has diffused throughout the orthopedic community, but CAN may become a secondary technology with the recent development of RAN [2]. The implementation of robotic systems has become increasingly common in medicine; furthermore, robotic systems have demonstrated accuracy in partial [2, 3] and total knee arthroplasty [1, 4]. RAN systems may also increase the level of standardization of care in arthroplasty procedures, which is associated with improved quality and efficiency of care [14]. 

Currently, there is little information comparing robotic to computer-assisted navigation technologies in TKA. The goal of this study was to compare the efficiency and accuracy of the two navigation systems. We compared navigation time and intraoperative final alignment of two commercially available systems and analyzed the bone cut accuracy of RAN. Our hypothesis is that the automaticity of the RAN system improves intraoperative navigation efficiency and alignment accuracy. To our knowledge, there is no existing clinical study comparing navigation times of these two types of systems.

## 2. Materials and Methods

### 2.1. Navigation Systems

**Figure 1:**
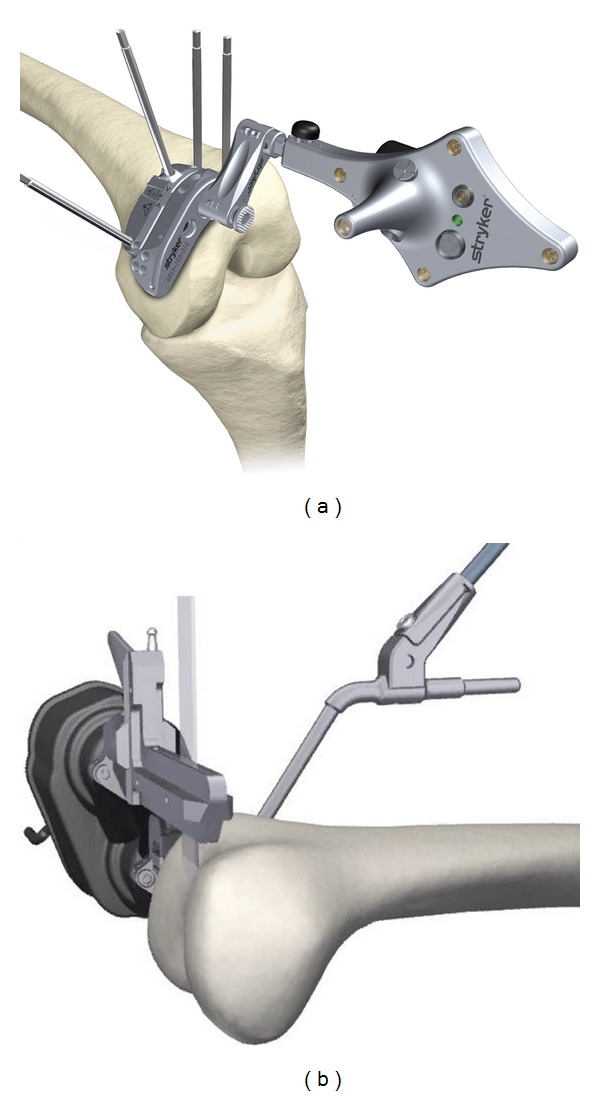
Femoral cutting blocks of the two navigation systems. (a) Stryker computer-assisted navigation. (b) Praxim robot-assisted navigation.

The two navigation systems used in this study were Stryker universal eNact knee v3.1 computer-assisted navigation (Stryker Kalamazoo, Michigan), Figure 1(a), and Praxim robot-assisted navigation (OMNIlife science, East Taunton, MA), Figure 1(b). The two systems share many similarities, but differ in critical aspects that influence their efficiency and accuracy. Both systems use imageless anatomic mapping of the knee and kinematic analysis of the limb to build a working model of the patient's knee. The Praxim System uses rigid bodies and probes that each have passive retroreflective markers that are localized in three-dimensional space by an optical infrared camera [15]. In contrast, the Stryker system uses light emitting diode navigation trackers [8]. The trackers/bodies are fixed to the distal femur and proximal tibia by cancellous bone screws. The camera can also track the position of a pointer or attachment and relate their positions to the mapped anatomy. 

The exact rotational center of the hip is acquired by rotating the limb in a circular motion. The pointer is then used to enter following anatomical landmarks: the femoral and tibia center, epicondylar axis, Whitesides line, rotational axis of tibia, and the malleoli of the ankle. The surgeon also uses the pointer to “paint” the entire distal surface of the femur; this is referred to as bone morphing [16]. The software then generates an accurate three-dimensional model of the patient's specific anatomy. Once the reference points are established, they allow the two systems to calculate the mechanical axis based on the absolute relationships of the patient's anatomy. The relationship of the navigation instruments to the patient's anatomy is shown intraoperatively in real-time and allows accurate planning of all bone cuts.

Before the cuts are executed, intraoperative kinematics analysis provides pathological varus and valgus angles producing real-time data for the surgeon [17]. Knee kinematics with trial components is then reviewed in real-time producing final intraoperative alignment data demonstrating if additional soft tissue release is necessary. 

The critical difference between the two systems is the automaticity of the RAN system. In addition, the RAN system provides component sizing to be performed with virtual assessment of notching. The robotic instrument is secured to the medial aspect of the distal femur using two cancellous bone screws [18]. The robotic arm automatically aligns a single cutting guide in the sagittal plane according to the surgeon verified plan for each of the following five femoral cuts: distal, anterior chamfer, anterior, posterior chamfer, and posterior (Figure 1(b)). Once the cuts are executed, a cut check is performed measuring the accuracy of the cut.

### 2.2. Data Source

After the Institution Review Board at the University of Washington approved this study and waived the requirement for patient consent, we examined electronic health records from Big Horn Basin Bone and Joint to identify TKA patients from 2006 to 2007 and 2010 to 2012. Big Horn Basin Bone and Joint is a private orthopaedic clinic located in Cody, Wyoming. 

### 2.3. Cohort Identification

After limiting our sample to patients in the correct time period, we identified a preliminary cohort of 100 patients who had undergone primary TKA with a preoperative diagnosis with one of the following: osteoarthritis, post-traumatic arthritis, or rheumatoid arthritis. Patients were excluded if there was preexisting hardware in the joint, if the procedure was performed at a different surgical institution, or if there was an absence of navigation data. In patients who had undergone bilateral primary TKA, data from only one knee were used (first knee of the two). We then divided the cohort into two groups based on the navigation type used. Group 1 consisted of primary TKAs performed between October 2010 and May 2012 with RAN. Group 2 consisted of primary TKAs performed between May 2006 and September 2007 with CAN. Patients with either varus or valgus deformities were included in the study and no patients were excluded based on excessive weight, age, or sex. This process yielded two groups consisting of 52 patients who underwent primary TKA utilizing RAN and 29 patients utilizing CAN. 

### 2.4. Operative Technique

One experienced orthopedic surgeon (Frank H. Schmidt) performed all of the TKAs at West Park Hospital (Cody, WY) in this study. The surgeon operated with one assistant in all cases. A medial parapatellar capsular incision was used in all knees. For all cases, the patella was resurfaced and the prosthetic components were cemented into place. Omni Apex PS prosthesis was used in the RAN group and DePuy Rotating platform prosthesis was used in the CAN group. 

### 2.5. Primary Outcome

The primary outcome of interest was navigation time. We analyzed the navigation data logs of each case to determine the total navigation time. The time recorded was measured from the start of the acquisition of the hip center to the end of the final alignment analysis for both systems.

### 2.6. Secondary Outcomes

Secondary outcomes included intraoperative final alignment, robot-assisted cut accuracy, tourniquet time, and hospitalization length. 

Final alignment was defined by the measurement of the intraoperative mechanical axis when the limb was at full extension and was obtained by reviewing the navigation report for each system that contained the quantitative measurement data of the procedure. The measurement indicates the intraoperative mechanical axis alignment of the prosthesis resulting in neutral (0° deviation) alignment, or a valgus or varus malalignment. 

Robot-assisted cut accuracy was defined by the difference between the planned and realized femoral flexion angle, femoral rotation angle, and tibia slope angle. The data was obtained by examining the navigation excel document containing the quantitative measurement data from the procedure. The femoral flexion cutting error was defined as the angular deviation in the sagittal plane of the distal femoral cut. The femoral rotation cutting error was defined as the deviation of the internal/external rotation for the anteroposterior cuts. The tibia slope cutting error was defined as the angular deviation in the sagittal plane of the tibia plateau cut. The system recorded the planned cutting plane before each cut and once the resections were performed, the cut surfaces were digitized recording the realized value. The values for the planned targets were defined as the numbers planned on the navigation system during the intraoperative virtual assessment prior to cut execution. The values for the executed results were defined as the numbers displayed by the navigation system when the cut controller was placed onto the resected surfaces. The amount of the cutting error with reference to the planned cutting plane in both the coronal plane and sagittal plane was measured by the navigation system.

Tourniquet time was defined as the time of inflation prior to incision to deflation immediately after the cement hardened for approximately 20 minutes. Hospitalization length was defined as the patients' admission into the hospital on the day of the procedure to when the patients were discharged.

### 2.7. Statistical Analysis

For the analysis of the collected data, a multivariate regression model was fit to each outcome of interest. Each outcome of interest was reported as an adjusted value to account for potential confounders through this regression model. The covariates for each multivariate regression model were carefully selected based on the assumption that none were affected directly by the intervention and were also individually fit to a univariate regression model to identify any association with the outcome of interest. These covariates included age, BMI, sex, prereplacement alignment, hypertension status, and intraoperative blood loss. All outcomes are adjusted associations with the respective intervention. *R* statistical software (version 2.15.1) was used to perform all analyses. Data are summarized using mean and standard deviation (SD) values and statistical significance was set at 0.05. 

## 3. Results

### 3.1. Baseline Characteristics

The baseline characteristics were similar with regard to age, sex, and BMI between the two groups and are summarized in Table 1. 

### 3.2. Primary Outcome

#### 3.2.1. Navigation Time

**Figure 2:**
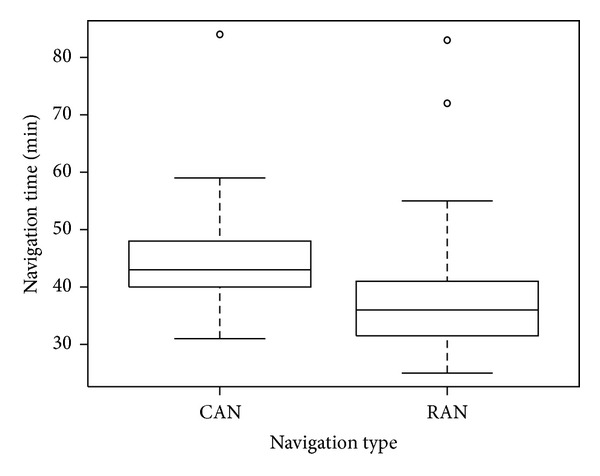
Boxplot of navigation times by navigation type.

We found navigation time to be significantly different between the two groups (*P* value = 0.0006). The RAN navigation times were, on average, 9.0 (95% CI:[4.0, 14.1]) minutes shorter when compared to CAN. This estimated difference is adjusted for age, BMI, and prereplacement alignment (Figure 2). 

### 3.3. Secondary Outcomes

#### 3.3.1. Final Alignment

We found the absolute intraoperative alignment to be significantly different between the two groups (*P* value = 0.022). The RAN alignments were, on average, 0.5 (95% CI:[0.07, 0.86]) degrees closer to the mechanical axis compared to CAN. This estimated difference is adjusted for age, BMI, and prereplacement alignment. Average final alignment for the RAN was 0.34° ± 0.67° valgus and 0.51° ± 0.69° varus. Average final alignment for the CAN system was 0.46° ± 0.43° valgus and 1.14° ± 1.14° varus. 

#### 3.3.2. Robot-Assisted Cut Accuracy

**Figure 3:**
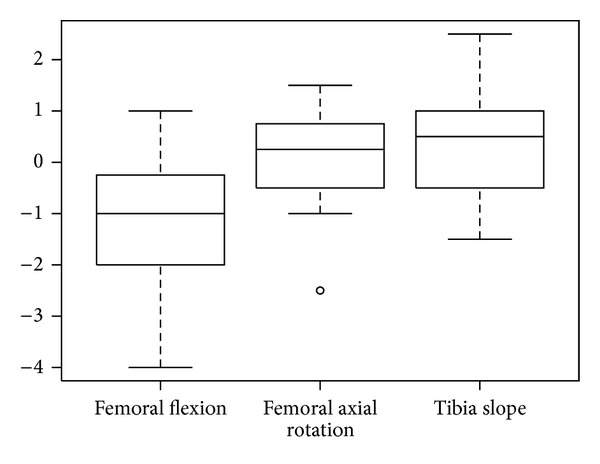
Boxplot of the mean cutting errors of RAN.

The femoral flexion mean cutting error was 1.1° ± 1.2°. The femoral axial rotation mean cutting error was 0.2° ± 0.8°. The tibia slope mean cutting error was 0.4° ± 1.0°. The distributions of the differences are shown in Figure 3. The realized femoral flexion angle tended to be less than planned, while the realized axial rotation and tibia slope angle tended to be higher than the planned value. Overall, 37% of the realized femoral cuts were within a half degree of the planned cut angle, 63% of axial rotations were within a half degree of the planned value, and 50% of the tibia slope cuts were within a half degree of the planned value. 

#### 3.3.3. Tourniquet Time

There was no statistically significant difference between the two systems: 0.2 (95% CI[5.4, 5.9], *P* value = 0.926). The estimated difference in tourniquet time between the two systems is adjusted for age, BMI, and hypertension status.

#### 3.3.4. Hospitalization Length

On average, patients in the RAN group were discharged 0.6 (95% CI:[0.1, 1.1]) days earlier compared to patients in the CAN group. The estimated difference in hospital stay length was statistically significant (*P* value: 0.0122), and was adjusted for age, BMI, and the amount of blood loss during surgery. 

## 4. Discussion

Comparative effectiveness research in TKA is needed because a significant percentage of the US population will undergo TKA [5, 9], and recently developed robot navigation systems have unknown efficiency and accuracy characteristics. This study demonstrates that continued technological innovation can increase efficiency and accuracy in TKA. We found navigation time to be significantly different between the two groups, with RAN navigation times being on average after adjustment, 9.0 minutes shorter when compared to CAN. We also found the absolute intraoperative alignment to be significantly different between the two groups. The RAN alignments were, on average, 0.5 degrees closer to the mechanical axis compared to CAN. Currently, it is difficult to compare our results because there are no similar clinical studies that we are aware of. 

 Differences in navigation time are a result of several factors. Femoral preparation time has been shown to be significantly less when using an automated cutting block compared to sequential cutting blocks [18]. There are also fewer navigation instruments associated the RAN system and may result in less navigation time by minimizing the time spent placing and handling the instruments. Also, the transitions between the five femoral cuts, when using the RAN system, are uninterrupted allowing seamless execution. The automaticity of the robotic instrument is likely the primary catalyst influencing the efficiency of the RAN system. 

The automaticity of the RAN system may also allow a standardization of TKA resulting in increased quality of patient care. The RAN system enables unprecedented accuracy of the distal femoral bone cuts while maintaining efficiency standards [18]. Bozic et al. concluded from an observational study that there is an association between greater hospital and procedure volume with shorter hospitalization length and fewer surgical complications. The authors also observed a strong correlation between overall process of care standardization and patient outcomes. They also concluded that process standardization could optimize quality and efficiency in TKA, independent of hospital or surgeon procedure volume [14]. This association between standardization of care and improved quality is important in underserved areas in the United States where patients do not have access to high volume tertiary care centers. Surgeons must have the ability to perform TKA with a standardized process utilizing automated navigation systems. 

In comparison to other studies, the RAN system achieved less cutting error than CAN systems. In a study conducted by Nakahara et al, the authors analyzed the distal femoral cuts using CAN. The authors demonstrated that the mean femoral flexion cutting error of all the knees in the study was 1.6° ± 2.2° [19]. RAN demonstrated 0.5° less cutting error accompanied by a 1° decrease in the standard deviation when compared to the CAN system used in the study. In comparison to another study by Yau and Chiu who measured the intraoperative cutting error using CAN, they reported a mean femoral flexion cutting error of 1.6° ± 1.3° and a mean tibia slope cutting error of 1.5° ± 0.8° [20]. Accurate execution of the distal femoral cuts is critical in the function and clinical outcome of the patient [21]. However, these are generalized comparisons and cannot be considered empirical, but they do allow the overall accuracy of RAN to be relatively quantified.

The tourniquet time did not coincide with the observed decrease in navigation time of RAN. This may be a result of several uncontrollable factors that influence tourniquet time. For example, two different types of bone cement were used to implant the components, and the cement used with the RAN system had a noticeably longer drying time, approximately 5–10 minutes longer, which is in the range of the difference observed in the navigation time. In addition, there may have been slight variations in wound irrigation or the experience of the operating room staff between the two time periods. Consequently, the variation of the surgical protocol between the two time periods likely influenced the tourniquet time and cannot be quantified or adjusted for in our statistical analysis. If a prospective study was conducted comparing RAN and CAN, we believe the tourniquet time would reflect the lower navigation time associated with the use of RAN. 

Our study had some limitations related to the use of the collected data. First, the systems were used for different lengths of time prior to the period of analysis for each group. The CAN system was used for approximately five years prior and the RAN system was not used prior to the period of analysis. Since there was a large cohort of RAN patients, the surgeon's proficiency likely normalized after the initial cases. A study conducted by. Jenny et al. observed a short learning curve for CAN system used in their study [22]. In another study by Sampath et al. the authors observed a similar trend but also observed continued performance improvement of the surgeons who used the CAN system [23], coinciding with our results. Therefore, the navigation times may differ substantially more than observed in our study. RAN's efficiency may increase over time resulting in significantly lower navigation times.

Second, our study was not a randomized trial, and patient comorbidities and age may have influenced the navigation time and accuracy of the two systems. Several studies have reported on postoperative femoral component alignment using radiographs or computed tomography [13, 17, 24], but no studies have evaluated the navigation times or the difference between the alignment of the cutting surface and that of the cutting guide intraoperatively using RAN. 

Third, our results suggest a statistically significant difference of final alignment between the two navigation systems. However, the rounding of the values for each of the two systems was not the same (CAN measurements were rounded to half-degrees, RAN measurements were rounded to degrees). After rounding CAN to degrees, the average difference was still closer to the mechanical axis for RAN, but the difference was no longer statistically significant (average difference: 0.3, 95% CI:[ 0.7, 0.2], *P* value: 0.211).

 Future studies will need to incorporate navigation time to correctly portray the effect of utilizing navigation systems when analyzing cost-effectiveness in TKA. The use of RAN allows the potential to standardize TKA, which may improve clinical outcomes. Additional studies need to be conducted evaluating the association between the precision of this system and clinical outcome. Technology offers opportunities to improve the quality of care delivered to patients and will be an integral part to patient care in the future but its efficacy must be validated. 

 In conclusion, our retrospective study found decreased navigation time and decreased intraoperative final malalignment was associated with the use of robot-assisted navigation compared to computer-assisted navigation independent of age, BMI, and prereplacement alignment. There was also a decreased hospitalization length associated with RAN use independent of age, BMI, and amount of blood loss during surgery.

## Figures and Tables

**Table 1:** Baseline characteristics, by navigation type.

Characteristic	No. (%)	
	RAN (*n* = 52)	CAN (*n* = 29)
Sex		
Men	21 (40)	10 (34)
Women	31 (60)	19 (66)
Race		
White	52 (100)	29 (100)
BMI—kg/m^2^ (SD)	33.5 (7.9)	30.2 (6.3)
Age, mean (SD)	66.1 (9.8)	68.3 (9.6)
Preoperative diagnosis		
Osteoarthritis	44 (85)	28 (97)
Post-traumatic arthritis	7 (13)	1 (3)
Rheumatoid arthritis	2 (4)	0 (0)
Comorbidities		
Diabetes mellitus	9 (21)	4 (14)
Essential hypertension	40 (77)	20 (69)
Past medical history		
Deep vein thrombosis	5 (10)	0 (0)
Pulmonary emboli	1 (2)	0 (0)
Stroke	1 (2)	1 (3)
Hx of smoking	11 (21)	5 (17)
Cardiovascular disease	13 (25)	13 (45)
Right knee	25 (48)	16 (55)
Left knee	27 (52)	13 (45)
Prereplacement malalignment (SD)	4.8 (2.9)	4.9 (3.3)
Prereplacement max extension (SD)	1.1 (4.0)	3.3 (3.5)

## References

[1] Lang JE, Mannava S, Floyd AJ (2011). Robotic systems in orthopaedic surgery. *Journal of Bone and Joint Surgery B*.

[2] Lonner JH, John TK, Conditt MA (2010). Robotic arm-assisted UKA improves tibial component alignment: a pilot study. *Clinical Orthopaedics and Related Research*.

[3] Pearle AD, O’Loughlin PF, Kendoff DO (2010). Robot-assisted unicompartmental knee arthroplasty. *Journal of Arthroplasty*.

[4] Bellemans J, Vandenneucker H, Vanlauwe J (2007). Robot-assisted total knee arthroplasty. *Clinical Orthopaedics and Related Research*.

[5] Iorio R, Robb WJ, Healy WL (2008). Orthopaedic surgeon workforce and volume assessment for total hip and knee replacement in the United States: preparing for an epidemic. *Journal of Bone and Joint Surgery A*.

[6] Gadinsky NE, Manuel JB, Lyman S, Westrich GH (2012). Increased operating room time in patients with obesity during primary total knee arthroplasty. Conflicts for scheduling. *Journal of Arthroplasty*.

[7] Huang NFR, Dowsey MM, Ee E, Stoney JD, Babazadeh S, Choong PF (2012). Coronal alignment correlates with outcome after total knee arthroplasty: five-year follow-up of a randomized controlled trial. *Journal of Arthroplasty*.

[8] Buehler KC (2008). Computer-assisted total knee arthroplasty: the state of the art in 2008—experience with Stryker Knee Nav in fixed bearing total knee arthroplasty. *Techniques in Knee Surgery*.

[9] Blakeney WG, Khan RJK, Wall SJ (2011). Computer-assisted techniques versus conventional guides for component alignment in total knee arthroplasty: a randomized controlled trial. *Journal of Bone and Joint Surgery A*.

[10] Chin PL, Yang KY, Yeo SJ, Lo NN (2005). Randomized control trial comparing radiographic total knee arthroplasty implant placement using computer navigation versus conventional technique. *Journal of Arthroplasty*.

[11] Hetaimish BM, Khan MM, Simunovic N, Al-Harbi HH, Bhandari M, Zalzal PK (2012). Meta-analysis of navigation versus conventional total knee arthroplasty. *Journal of Arthroplasty*.

[12] Choong PF, Dowsey MM, Stoney JD (2009). Does accurate anatomical alignment result in better function and quality of life? Comparing conventional and computer-assisted total knee arthroplasty. *Journal of Arthroplasty*.

[13] Zhang G, Chen J, Chai W, Liu M, Wang Y (2011). Comparison between computer-assisted-navigation and conventional total knee arthroplasties in patients undergoing simultaneous bilateral procedures: a randomized clinical trial. *Journal of Bone and Joint Surgery A*.

[14] Bozic KJ, Maselli J, Pekow PS, Lindenauer PK, Vail TP, Auerbach AD (2010). The influence of procedure volumes and standardization of care on quality and efficiency in total joint replacement surgery. *Journal of Bone and Joint Surgery A*.

[15] Plaskos C, Cinquin P, Lavallée S, Hodgson AJ (2005). Praxiteles: a miniature bone-mounted robot for minimal access total knee arthroplasty. *The International Journal of Medical Robotics and Computer Assisted Surgery*.

[16] Koulalis D, O’Loughlin PF, Plaskos C, Kendoff D, Pearle AD (2010). Adjustable cutting blocks for computer-navigated total knee arthroplasty. A cadaver study. *Journal of Arthroplasty*.

[17] Harvie P, Sloan K, Beaver RJ (2011). Three-dimensional component alignment and functional outcome in computer-navigated total knee arthroplasty. A prospective, randomized study comparing two navigation systems. *Journal of Arthroplasty*.

[18] Koulalis D, O’Loughlin PF, Plaskos C, Kendoff D, Cross MB, Pearle AD (2011). Sequential versus automated cutting guides in computer-assisted total knee arthroplasty. *Knee*.

[19] Nakahara H, Matsuda S, Moro-oka T, Okazaki K, Tashiro Y, Iwamoto Y (2012). Cutting error of the distal femur in total knee arthroplasty by use of a navigation system. *Journal of Arthroplasty*.

[20] Yau WP, Chiu KY (2008). Cutting errors in total knee replacement: assessment by computer assisted surgery. *Knee Surgery, Sports Traumatology, Arthroscopy*.

[21] Anglin C, Brimacombe JM, Hodgson AJ (2008). Determinants of patellar tracking in total knee arthroplasty. *Clinical Biomechanics*.

[22] Jenny J, Miehlke RK, Giurea A (2008). Learning curve in navigated total knee replacement. A multi-centre study comparing experienced and beginner centres. *Knee*.

[23] Sampath SAC, Voon SH, Sangster M, Davies H (2009). The statistical relationship between varus deformity, surgeon’s experience, BMI and tourniquet time for computer assisted total knee replacements. *Knee*.

[24] Huang TW, Hsu WH, Peng KT, Hsu RW, Weng YJ, Shen WJ (2011). Total knee arthroplasty with use of computer-assisted navigation compared with conventional guiding systems in the same patient: radiographic results in asian patients. *Journal of Bone and Joint Surgery A*.

